# A Case of Warthin-Like Variant of Papillary Thyroid Cancer

**DOI:** 10.7759/cureus.91182

**Published:** 2025-08-28

**Authors:** Amy Chow, Israa Laklouk

**Affiliations:** 1 Endocrinology, University of California Los Angeles David Geffen School of Medicine, Thousand Oaks, USA; 2 Pathology, University of California Los Angeles David Geffen School of Medicine, Los Angeles, USA

**Keywords:** benign and malignant thyroid nodule, braf v600e mutations, head and neck mass, papillary carcinoma of thyroid, thyroid carcinoma, warthin-like variant of papillary thyroid carcinoma

## Abstract

Warthin-like variant of papillary thyroid cancer (WLPTC) is a rare subtype of papillary thyroid cancer (PTC). Its symptoms, diagnosis, treatment, and prognosis are comparable to those of the classic type of PTC. However, certain aggressive PTC variants may mimic WLPTC, making accurate diagnosis essential since management and follow-up can differ. Here, we report a case of a 64-year-old man who was diagnosed with WLPTC. He initially presented to the hospital with worsening blurry vision of the right eye over a few days. During evaluation, an incidental right neck mass was detected, and fine-needle aspiration was performed. The pathology revealed thyroid carcinoma. The patient subsequently underwent total thyroidectomy with modified neck dissection, and histological analysis identified WLPTC. Postoperatively, he received radioactive iodine therapy and thyroid hormone therapy. His follow-up visits showed a favorable outcome two years later. This case highlights the importance of identifying WLPTC, as accurate diagnosis is crucial for ensuring appropriate clinical management and follow-up for patients.

## Introduction

Papillary thyroid cancer (PTC) is the most prevalent type of thyroid carcinoma, and it has several different variants [1]. Tall cell, follicular, and classical variants are the most common types [2]. Among these, the tall cell variant has a poorer prognosis compared to others [3]. The Warthin-like variant of PTC, considered a subtype of the oncocytic variant, is a rare form that accounts for about 0.2-1.9% of all PTC cases [4]. Warthin-like variant of PTC (WLPTC) was first described in 1995 [5]. The name was suggested by Apel et al. because its histological features closely resemble Warthin tumor of the salivary gland [5]. To date, about 200 cases have been described in the English literature [6,7].

WLPTC primarily affects women and is most often diagnosed in younger individuals, typically between 30 and 50 years of age [3]. Its prognosis is similar to that of the classical variant of PTC [3]. However, cytological diagnosis can be challenging due to overlapping features with other PTC variants [7]. Accurate differential diagnosis is critical, as certain mimics, such as the tall cell variant, are associated with more aggressive behavior and less favorable outcomes compared to WLPTC [7]. This case report highlights the clinicopathologic features of WLPTC and emphasizes the importance of accurate diagnosis for optimal patient management and follow-up.

## Case presentation

A 64-year-old man without prior history of thyroid disease presented to the emergency room with worsening blurry vision in his right eye over several days. He underwent extensive evaluations during hospitalization. Lumbar puncture results were negative for infectious, neoplastic, or autoimmune causes. MRI of the orbits showed atypical optic neuritis, which resolved after five days of intravenous steroid therapy. MRI of the cervical, thoracic, and lumbar spine was also performed. His cervical MRI with and without contrast showed an incidental 2.5 cm right neck mass, likely an enlarged level II/III lymph node compressing the right internal jugular vein. Thyroid ultrasound showed the right lobe measured 4.49 cm × 1.4 cm × 1.76 cm. A right superior hypoechoic solid nodule measuring 1.11 cm × 0.61 cm × 0.85 cm was identified, causing anterior bulging of the capsule and demonstrating internal punctate microcalcifications. This nodule had a smooth margin and was classified as TI-RADS level 5 (TR5) [8]. There was a round, isoechoic right level III lymph node measuring 3.08 cm × 1.66 cm × 2.91 cm that contained internal coarse calcifications. The left lobe measured 5.01 cm × 1.44 cm × 1.51 cm and contained no nodules. The isthmus measured 0.29 cm and contained no nodules. His TSH level was within the normal range on admission. Thyroid peroxidase antibody and thyroglobulin antibody were negative for Hashimoto’s thyroiditis. The patient had no prior radiation exposure or family history of thyroid cancer.

**Figure 1:**
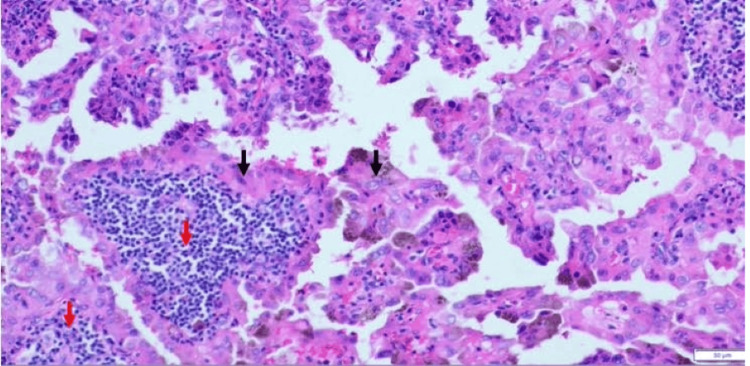
H&E stain, 200X. Warthin-like papillary thyroid carcinoma (WLPTC) showing papillary lining of neoplastic cells with eosinophilic cytoplasm (black arrows) and abundant lymphoid stroma (red arrows), resembling Warthin tumors of the salivary gland.

**Figure 2:**
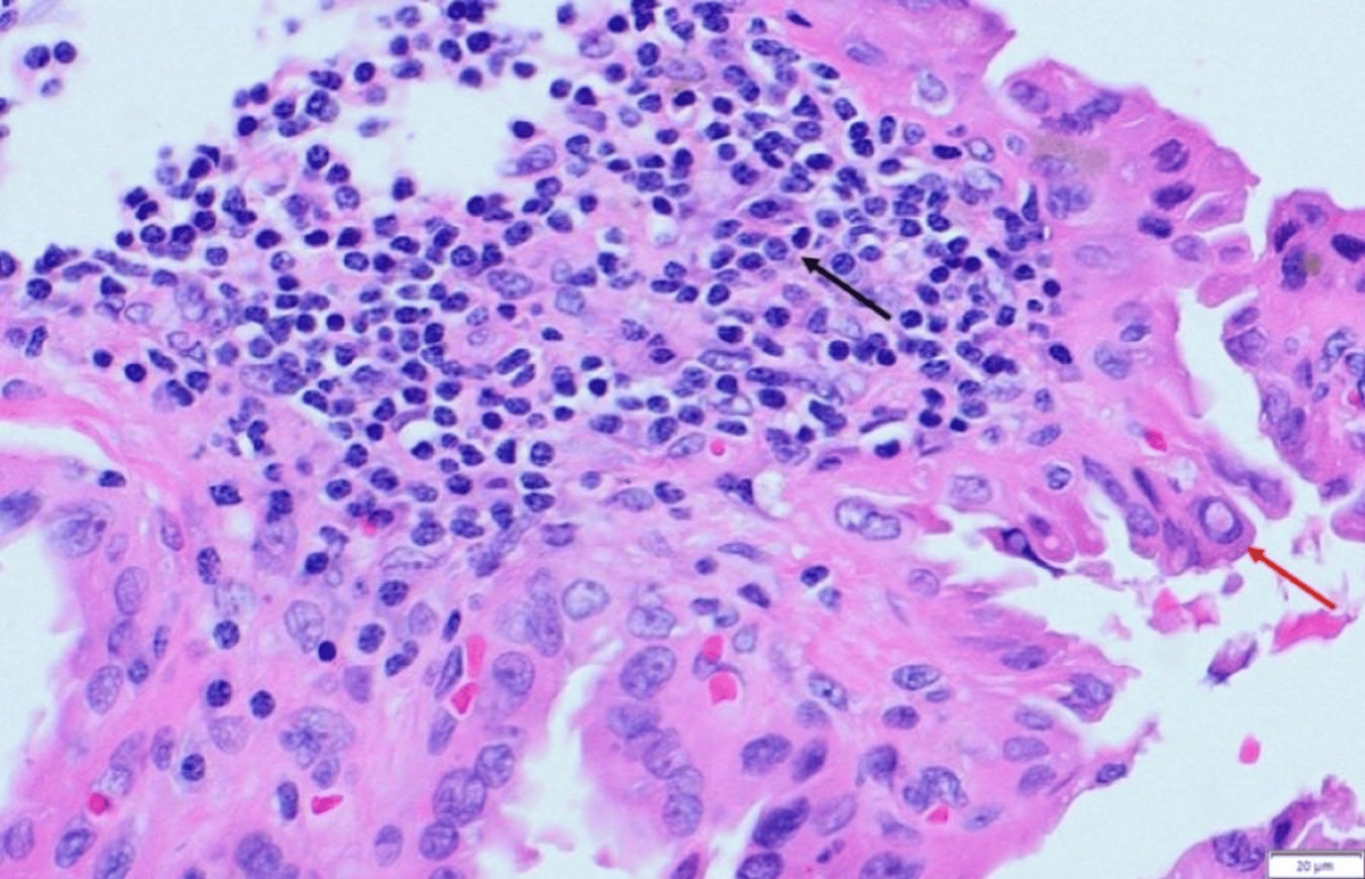
H&E stain, 400X. High-power view showing neoplastic oncocytic cells with papillary thyroid carcinoma (PTC) nuclear features, including nuclear clearing, pseudoinclusions (red arrow), grooves, and nuclear overlap, within a lymphoplasmacytic-rich fibrovascular stroma (black arrow).

The right thyroid nodule was not biopsied; therefore, no mutation study was performed. However, he underwent fine-needle aspiration of the right level III lymph node, and the pathology showed metastatic carcinoma consistent with thyroid origin. The patient subsequently underwent total thyroidectomy with modified radical neck dissection. Surgical pathology confirmed a 0.8 cm Warthin-like subtype papillary thyroid carcinoma in the right thyroid lobe (Figures 1, 2).

There was evidence of lymphatic invasion. No thyroid carcinoma was identified in the left lobe. Papillary thyroid carcinoma was present in one of eleven lymph nodes at levels III and IV of the right neck, with a tumor deposit measuring 2.8 cm in maximum dimension. Papillary thyroid carcinoma was also present in one of two lymph nodes at level II of the right neck, with a tumor deposit measuring 4 mm. No extranodal extension was identified in the metastatic lymph nodes.

The patient was treated with iodine-131 ablation and suppressive thyroxine therapy. The two-year outcomes were favorable.

## Discussion

WLPTC is a rare oncocytic variant of PTC, predominantly affecting women [3]. It commonly presents as a painless neck mass [3]. Ultrasound of the neck usually reveals a solid, hypoechoic nodule with heterogeneous parenchyma in the background, but it may be misdiagnosed as a benign nodule or focal thyroiditis [9]. A case series of 150 patients with WLPTC showed thyroid autoimmunity was positive in 71.6% of cases, and lymph node metastases were present in 20%, with no reported distant metastases [10]. Our patient had no autoimmune thyroid disease, which is atypical for WLPTC. His thyroid ultrasound showed a TR5 thyroid nodule with features concerning for malignancy. Surgical pathology confirmed lymph node metastasis, aligning with other reported cases.

There are no specific clinical or radiological findings for diagnosing WLPTC. Fine-needle aspiration is required if a painless neck mass is found on ultrasound [3]. However, fine-needle biopsy results can be inconclusive [3]. Histopathological assessment of thyroidectomy specimens is crucial for a definitive diagnosis [3]. Histologically, WLPTC may present with either a well-circumscribed or infiltrative growth pattern [11]. On low-power examination, its morphology resembles that of a Warthin tumor of the salivary gland [5]. This resemblance stems from the presence of arborizing papillary structures lined by oncocytic cells, which exhibit the classic nuclear features of PTC (nuclear overlapping, irregular contours, grooves, pseudoinclusions, and clearing) [11]. The papillary cores are typically rich in lymphocytes and plasma cells, contributing to the tumor’s distinctive appearance [7]. This prominent lymphoplasmacytic infiltrate is a hallmark of the Warthin-like variant and aids in distinguishing it from other PTC subtypes [11].

The differential diagnosis of WLPTC includes Hashimoto’s thyroiditis, Hürthle cell neoplasm, classical subtype PTC arising in a thyroiditis background, tall cell subtype, and oncocytic subtype of PTC [3]. Differentiating WLPTC from other PTC subtypes is critical, as some, such as the tall cell variant, have more aggressive behavior and require different management [7]. Key features that distinguish WLPTC from the tall cell variant include: WLPTC papillary cores have dense lymphocytic infiltrates, whereas tall cell variant papillary cores have very few lymphocytic infiltrates [7]. WLPTC is less likely to show lymphovascular invasion or distant metastasis compared to the tall cell variant, which is associated with worse outcomes [7]. The above features help distinguish between these two variants. Our patient’s histological analysis, which showed dense lymphocytic infiltrates, supported the diagnosis of WLPTC.

Immunohistochemically, the BRAFV600E mutation is the most common mutation and may play a role in the pathogenesis of classic PTC [12]. These immunohistochemical markers are not known to play a significant role in differential pathological diagnosis of WLPTC [3]. In this case, BRAF mutation testing was not performed.

Treatment typically involves surgery (lobectomy or total thyroidectomy), with possible neck dissection depending on the tumor’s extent [13]. Postoperative radioactive iodine ablation may be considered for higher-stage cases [13]. WLPTC generally has a favorable prognosis, with low rates of recurrence, extrathyroidal extension, and distant metastasis [14]. Our patient, despite having lymph node metastasis, has been doing well for two years post-treatment.

## Conclusions

WLPTC is a rare and often indolent variant of PTC, requiring careful histopathological evaluation for accurate diagnosis. Its distinctive features, such as the dense lymphocytic infiltrate in papillary cores, help distinguish it from more aggressive PTC subtypes like the tall cell variant. Although treatment usually involves surgery and possible adjuvant therapies, WLPTC generally follows a favorable clinical course, emphasizing the importance of correct diagnosis for optimal management.
